# Expanding Disease Definitions in Guidelines and Expert Panel Ties to Industry: A Cross-sectional Study of Common Conditions in the United States

**DOI:** 10.1371/journal.pmed.1001500

**Published:** 2013-08-13

**Authors:** Raymond N. Moynihan, Georga P. E. Cooke, Jenny A. Doust, Lisa Bero, Suzanne Hill, Paul P. Glasziou

**Affiliations:** 1 Bond University, Robina, Australia; 2 University of California, San Francisco, San Francisco, California, United States of America; 3 Australian National University, Acton, Australia; Harvard University, Brigham and Women's Hospital, United States of America

## Abstract

**Background:**

Financial ties between health professionals and industry may unduly influence professional judgments and some researchers have suggested that widening disease definitions may be one driver of over-diagnosis, bringing potentially unnecessary labeling and harm. We aimed to identify guidelines in which disease definitions were changed, to assess whether any proposed changes would increase the numbers of individuals considered to have the disease, whether potential harms of expanding disease definitions were investigated, and the extent of members' industry ties.

**Methods and Findings:**

We undertook a cross-sectional study of the most recent publication between 2000 and 2013 from national and international guideline panels making decisions about definitions or diagnostic criteria for common conditions in the United States. We assessed whether proposed changes widened or narrowed disease definitions, rationales offered, mention of potential harms of those changes, and the nature and extent of disclosed ties between members and pharmaceutical or device companies.

Of 16 publications on 14 common conditions, ten proposed changes widening and one narrowing definitions. For five, impact was unclear. Widening fell into three categories: creating “pre-disease”; lowering diagnostic thresholds; and proposing earlier or different diagnostic methods. Rationales included standardising diagnostic criteria and new evidence about risks for people previously considered to not have the disease. No publication included rigorous assessment of potential harms of proposed changes.

Among 14 panels with disclosures, the average proportion of members with industry ties was 75%. Twelve were chaired by people with ties. For members with ties, the median number of companies to which they had ties was seven. Companies with ties to the highest proportions of members were active in the relevant therapeutic area. Limitations arise from reliance on only disclosed ties, and exclusion of conditions too broad to enable analysis of single panel publications.

**Conclusions:**

For the common conditions studied, a majority of panels proposed changes to disease definitions that increased the number of individuals considered to have the disease, none reported rigorous assessment of potential harms of that widening, and most had a majority of members disclosing financial ties to pharmaceutical companies.

*Please see later in the article for the Editors' Summary*

## Editors' Summary

**Background:**

Health professionals generally base their diagnosis of physical and mental disorders among their patients on disease definitions and diagnostic thresholds that are drawn up by expert panels and published as statements or as part of clinical practice guidelines. These disease definitions and diagnostic thresholds are reviewed and updated in response to changes in disease detection methods, treatments, medical knowledge, and, in the case of mental illness, changes in cultural norms. Sometimes, the review process widens disease definitions and lowers diagnostic thresholds. Such changes can be beneficial. For example, they might ensure that life-threatening conditions are diagnosed early when they are still treatable. But the widening of disease definitions can also lead to over-diagnosis—the diagnosis of a condition in a healthy individual that will never cause any symptoms and won't lead to an early death. Over-diagnosis can unnecessarily label people as ill, harm healthy individuals by exposing them to treatments they do not need, and waste resources that could be used to treat or prevent “genuine” illness.

**Why Was This Study Done?:**

In recent years, evidence for widespread financial and non-financial ties between pharmaceutical companies and the health professionals involved in writing clinical practice guidelines has increased, and concern that these links may influence professional judgments has grown. As a result, a 2011 report from the US Institute of Medicine (IOM) recommended that, whenever possible, guideline developers should not have conflicts of interest, that a minority of the panel members involved in guideline development should have conflicts of interest, and that the chairs of these panels should be free of conflicts. Much less is known, however, about the ties between industry and the health professionals involved in reviewing disease definitions and whether these ties might in some way contribute to over-diagnosis. In this cross-sectional study (an investigation that takes a snapshot of a situation at a single time point), the researchers identify panels that have recently made decisions about definitions or diagnostic thresholds for conditions that are common in the US and describe the industry ties among the panel members and the changes in disease definitions proposed by the panels.

**What Did the Researchers Do and Find?:**

The researchers identified 16 publications in which expert panels proposed changes to the disease definitions and diagnostic criteria for 14 conditions that are common in the US such as hypertension (high blood pressure) and Alzheimer disease. The proposed changes widened the disease definition for ten diseases, narrowed it for one disease, and had an unclear impact for five diseases. Reasons included in the publications for changing disease definitions included new evidence of risk for people previously considered normal (pre-hypertension) and the emergence of new biomarkers, tests, or treatments (Alzheimer disease). Only six of the panels mentioned possible harms of the proposed changes and none appeared to rigorously assess the downsides of expanding definitions. Of the 15 panels involved in the publications (one panel produced two publications), 12 included members who disclosed financial ties to multiple companies. Notably, the commonest industrial ties among these panels were to companies marketing drugs for the disease being considered by that panel. On average, 75% of panel members disclosed industry ties (range 0% to 100%) to a median of seven companies each. Moreover, similar proportions of panel members disclosed industry ties in publications released before and after the 2011 IOM report.

**What Do These Findings Mean?:**

These findings show that, for the conditions studied, most panels considering disease definitions and diagnostic criteria proposed changes that widened disease definitions and that financial ties with pharmaceutical companies with direct interests in the therapeutic area covered by the panel were common among panel members. Because this study does not include a comparison group, these findings do not establish a causal link between industry ties and proposals to change disease definitions. Moreover, because the study concentrates on a subset of common diseases in the US setting, the generalizability of these findings is limited. Despite these and other study limitations, these findings provide new information about the ties between industry and influential medical professionals and raise questions about the current processes of disease definition. Future research, the researchers suggest, should investigate how disease definitions change over time, how much money panel members receive from industry, and how panel proposals affect the potential market of sponsors. Finally it should aim to design new processes for reviewing disease definitions that are free from potential conflicts of interest.

**Additional Information:**

Please access these Web sites via the online version of this summary at http://dx.doi.org/10.1371/journal.pmed.1001500.

- A *PLOS Medicine* Research Article by Knüppel et al.  assesses the representation of ethical issues in general clinical practice guidelines on dementia care
- Wikipedia has a page on medical diagnosis (note: Wikipedia is a free online encyclopedia that anyone can edit; available in several languages)
- An article on over-diagnosis by two of the study authors is available; an international conference on preventing over-diagnosis will take place this September
- The 2011 US Institute of Medicine report Clinical Practice Guidelines We Can Trust is available
- A *PLOS Medicine* Essay by Lisa Cosgrove and Sheldon Krimsky discusses the financial ties with industry of panel members involved in the preparation of the latest revision of the American Psychiatric Association's *Diagnostic and Statistical Manual of Mental Disorders* (*DSM*), which provides standard criteria for the classification of mental disorders

## Introduction

Changes in technologies, treatments, medical knowledge, and cultural norms provide cause to review and change disease definitions and diagnostic thresholds, a task that is commonly undertaken by expert panels, consensus meetings, or influential workgroups who publish findings as statements, special reports, or as part of clinical practice guidelines. While such changes can be beneficial, there is an increasing recognition that widening of disease definitions may be one factor contributing to the problem of over-diagnosis, occurring across a range of conditions including pulmonary embolism, breast and prostate cancers [1],[2]. The concern expressed by some researchers is that for some people with milder symptoms, at lower risks, or in earlier stages of possible disease, the harms of a diagnostic label and treatment may outweigh benefits [3],[4].

At the same time there is accumulating evidence about pervasive financial ties between pharmaceutical companies and health professionals [5], including those writing guidelines [6] and disease definitions [7]. While noting the value of professional–industry collaborations, a 2009 Institute of Medicine (IOM) report found “widespread relationships with industry have created significant risks that individual and institutional financial interests may unduly influence professionals' judgments,” and that these “conflicts of interest” threaten the integrity of research, the objectivity of education, the quality of patient care, and public trust in medicine [5].

The 2009 report recommended professional societies and other organisations drafting clinical practice guidelines should “generally exclude as panel members individuals with conflicts of interest.” A subsequent 2011 IOM report on how to produce trustworthy guidelines included recommendations that “whenever possible,” guideline developers “should not have” conflicts of interest, that only a minority should have conflicts, and that chairs should be free of conflicts [8].

As both reports make clear, in addition to financial ties there are non-financial or intellectual conflicts such as academic advancement, and there should be no assumption that having a conflict is unethical, or “that any particular professional will necessarily let financial gain influence his or her judgment” [5].

A 2011 systematic review found many clinical guideline panels have failed to disclose financial ties, and those that did disclose had a “high percentage” of individuals with financial conflicts of interest [6]. Studies analysing ties of working groups for the Diagnostic and Statistical Manual of Mental Disorders (DSM), which set definitions and diagnostic criteria, have also found a majority of members with ties [7]. Kung and colleagues recently found two-thirds of individuals chairing guideline committees had conflicts of interest [9].

Few studies [7] have examined the financial ties of members of panels reviewing and changing definitions of common conditions, whether as part of practice guideline development or other processes. Our aim was to identify guideline panels in the US setting that have most recently made decisions about definitions or diagnostic thresholds for common conditions, and to report on any proposed changes and their industry ties.

## Methods

### List of Conditions

**Figure 1:**
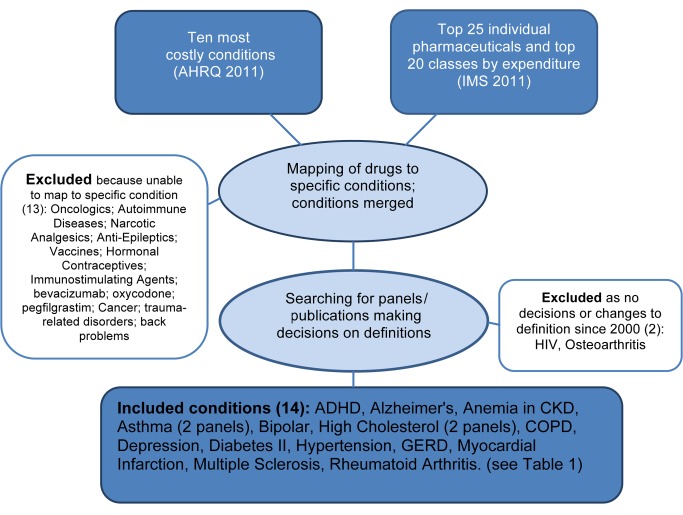
Flowchart identifying study conditions and panels reviewing definitions. Note: bipolar/depression was one panel.

On the basis of the method previously used by Choudhry and colleagues [10], we derived a list of common conditions in the United States, drawing from a list of the ten most costly adult diseases [11], the top 20 therapeutic classes of drugs, and the top 25 individual drugs by expenditure [12]. Consistent with that method, drugs used to treat many non-specific conditions were excluded (e.g., pain killers). For situations in which a drug was approved for a number of conditions, we identified the most common condition for inclusion (e.g., etanercept ultimately mapped to rheumatoid arthritis, not psoriatic arthritis). If a condition in the top ten costly disease list was too broad or diffuse, or covered many specific conditions, it was excluded (e.g., back problems). A flowchart of the method appears in Figure 1.

### List of Panels and Publications

We aimed to identify the most recent publication from panels making decisions about disease definition and diagnosis. A panel publication was eligible for inclusion if it was generated or supported by a widely recognised US-based organisation, published between 2000 and April 2013, and included deliberations and decisions on disease definitions and/or diagnostic criteria, classification, or assessment. If the panel made decisions, but proposed no changes, our search would continue for the most recent publication proposing changes, to include as well. If the focus of the panel publication was limited to specific sub-groups of patients, (e.g., adolescents), specific sub-categories of the condition (e.g., work-related asthma), it came from a single entity (e.g., a health maintenance organisation), or it included treatment recommendations but no review and deliberation on disease definition or diagnostic criteria, it was excluded.

During a pilot phase, using the searches for the most recent hypertension and asthma panel publications, an explicit search strategy using standardized keywords was iteratively developed in order to maximise sensitivity. We searched Medline (Ovid) using terms for each disease/condition and combined these terms with a standardized search strategy consisting of a string of MeSH and keyword terms to identify panels and publications (example in Table S1). Searches were run over 26–31 July 2012, updated 17–18 April 2013, and limited to English language from 2000.

To further improve sensitivity and try to ensure recent publications were not missed, two authors (RNM, GPEC) independently analysed the results of the standardised Medline searches for all conditions, and supplemented this with independent individual searches of the websites of the relevant National Institutes of Health and the National Guideline Clearing House. For two conditions, minor discrepancies in independent suggestions were resolved by discussion and, in one case (diabetes II), by consultation with a third author (PPG). Because of their global prominence and influence, if a panel was constituted under the umbrella of the National Institutes of Health (NIH), or the American Psychiatric Association's DSM, and met our inclusion criteria, these panels were identified for inclusion in our study. If there was a more recent panel publication that also met the study's inclusion criteria, in addition to the NIH or DSM panel, we included the more recent publication as well. This occurred twice (asthma and high cholesterol), resulting in two publications being identified for each condition.

### Information on the Panels' Decisions

For each publication we extracted information on key proposed changes to definitions/diagnostic criteria, the rationale offered, and any mention of potential harms associated with the proposed changes (e.g., over-diagnosis, overtreatment, medicalizing normality, labelling asymptomatic people). All six authors then made an assessment of whether the panel's proposed key changes would tend to widen (e.g., earlier diagnosis, lower thresholds, adding symptoms, increasing numbers diagnosed) or narrow the disease definition, or whether it was unclear.

### Information on Industry Ties

Using published disclosure sections from the panel publications, duplicate independent extraction of data was conducted (RNM and research assistant Peter Coxeter), with a third party resolving any disagreement (PGG). Ties were categorized as speaker/honorarium, consultant/adviser, grant/research, stock, employee, travel, or royalties. Panel members were those listed as authors or identified as the group with primary responsibility for generating the publications. In line with the IOM approach [5], an industry tie was defined as a tie to a pharmaceutical, diagnostic, device, or biotechnology company, but not a communications or medical education company. If there was any lack of clarity as to the nature of the company, or uncertainty if it met study criteria, a tie was not recorded. Once all industry ties were recorded for each panel, websites of companies with financial ties to the three highest proportions of panel members were searched to determine whether those companies were active in the specific therapeutic area. Where they appeared in disclosure sections, the disclosure of any ties to public agencies, non-government organizations, and publishers was also recorded.

## Results

After analyzing source documents [11],[12], the following drug classes, individual drugs, and conditions were excluded when identifying study conditions, as they were too non-specific or too broad, and did not map to specific conditions enabling analysis: oncologics; autoimmune diseases; narcotic analgesics; anti-epileptics; vaccines; hormonal contraceptives; immunostimulating agents; bevacizumab; oxycodone; pegfilgrastim; cancer; trauma-related disorders; and back problems.

From an initial list of 16 included common conditions, for two—osteoarthritis, HIV—we could identify no panel that made decisions about definitions or diagnostic thresholds since 2000 in the US context specifically. For the remaining 14 conditions, we identified the most recent panels that deliberated and made decisions about disease definitions, all of which proposed changes. For asthma and high cholesterol we identified two panels each, one constituted under the government funded NIH [13],[14] and one by professional societies [15],[16], reflecting the two main types of panels identified in this study. A single panel, the DSM-V Mood Disorders working group, proposed changes to two different conditions, bipolar and depression, in two separate web-based publications [17]. A full list of the final 14 conditions, 15 panels and 16 publications, key changes and rationale, analysis of panel decisions, and disclosed ties appears in Table 1.

**Table 1:** Conditions and characteristics of the panels and publications included in the study.

Disease	Panel	Change/s	Rationale	Widens or Narrows?	Mentions Risk?	Discloses?	Ties?	Chair Ties?	For Members with Ties, Mean Numbers:			Percent Members Disclosing Non-Industry Ties
									Speaker/Honoraria Ties	Consultant/Adviser Ties	Research/Grant Ties	
ADHD	DSM V 2012 [19]	Changes onset age; expands symptoms	Help facilitate adult diagnosis; previous age invalid	Widen	y	y	y	n	2	1.2	1.8	78
Alzheimer	NIA-AA 2011 [28]–[30]	Creates new categories	Update; new evidence about biomarkers	Widen	y	y	y	y	0	4.5	0.9	13
Anemia/CKD	KDIGO 2012 [33]	Narrows definition	In line with WHO	Narrow	n	y	y	y	0.9	2.3	1.2	n/a
Asthma	ATS/ERS 2009 [15]	Redefines “severity”	Complex	Unclear	n	y	y	y	2.9	5.3	4.3	13
Asthma	EPR 3 2007 [13]	New classification; removes “mild-intermittent”	Complex	Unclear	unclear	y	y	y	3.3	6.4	4.1	39
Bipolar	DSM V 2012 [17]	Adds core symptom, new sepcifier	Complex	Unclear	y	y	y	y	2.1	3	1.8	83
Depression^a^	DSM V 2012	Removes bereavement exclusion	Exclusion was not justified	Widen	y							
Cholesterol^b^	ATP III 2002 [14]	Changes thresholds	Risk of future events	Widen	n	n/a	n/a	n/a	n/a	n/a	n/a	n/a
Cholesterol	AACE 2012 [16]	Recommends additional new test	Evidence test can predict risk	Widen	n	y	y	y	3.6	2.1	1.4	n/a
COPD^c^	GOLD 2011 [18]	Changes diagnostic method and classification	Simplicity; old system inadequate	Unclear	y	y	y	y	n/a	n/a	n/a	8
Diabetes II^d^	International Expert Committee 2009 [22]	Changes diagnostic method; new cut-points	Better test; cut-point related to future risk	Unclear	y	y	n	n	n/a	n/a	n/a	n/a
GERD^e^	Montreal Definition 2006 [21]	New definition and classification	Better for research, simplify management	Widen	n	y	y	y	n/a	n/a	n/a	n/a
Hypertension	JNC7 2003 [23]	Creates new diagnostic category	Risk of future complications	Widen	n	y	y	y	4.9	6.7	6.9	27
MS	2010 Revision McDonald Criteria [42]	Changes imaging criteria for diagnosis	Simplify diagnosis, reduced testing	Widen	n	y	y	y	2.6	6.1	2.7	83
MI^f^	Universal Definition 2012 [20]	Changes to criteria and classification	Development of more sensitive tests	Widen	y	y	y	y	n/a	n/a	n/a	21
RA^g^	ACR/ELARCI 2010 [43]	New classification system	Early intervention; consistency in research	Widen	n	y	y	y	n/a	n/a	n/a	3

a The same Mood Disorders work group proposed separate changes to bipolar and depression.

b The cholesterol 2002 panel publication was silent on disclosure.

c The COPD panel did not separate speaker and consultant ties.

d The diabetes panel disclosed no ties.

e GERD panel disclosures only pertained to one company and did not include separate categories.

f The myocardial infarction panel reported ties in a method that did not allow categorization of different forms of tie.

g The rheumatoid arthritis panel did not separate all speaker and consultant ties.

MI, myocardial infarction; MS, multiple sclerosis; n/a, not available; RA, rheumatoid arthritis.

Among 16 publications, all authors in our study agreed that proposals in ten publications would tend to widen definitions (Table 2) and for one, narrow the definition. For the remaining five publications the impact was unclear. Rationales for the benefits of widening definitions or expanding diagnostic categories included: evidence about the risk of future adverse events for people previously considered normal (pre-hypertension); simplification (gastroesophageal reflux disease [GERD]); standardisation for research (rheumatoid arthritis); and the emergence of new evidence about biomarkers, tests, or treatments (Alzheimer disease). Among 15 panels, six included mention of possible harms of proposed changes (Table 3), albeit briefly, with three of those including citations in that mention [17]–[19], two citing primary studies [18],[19], and one of those citing a review of primary studies as well [18]. One publication referred to the potential negative consequences for those who would be labelled by the expanded definition [20], and only one referred to overdiagnosis [21].

**Table 2:** Different ways to expand disease definitions.

Method of Widening	Disease	Details
Creating new categories of pre-disease	Hypertension	Describes pre-hypertension
	Alzheimer disease	Describes pre-dementia and defines pre-clinical Alzheimer disease
Lowering diagnostic thresholds	High cholesterol 2002	Lowers cholesterol and triglyceride thresholds
	ADHD	Changes age of onset; adds new symptoms
	Depression	Removes bereavement exclusion
	GERD	Drops severity threshold for definition
Earlier diagnosis, different diagnostic method	Rheumatoid arthritis	Earlier diagnosis
	Multiple sclerosis	Single scan diagnosis, earlier identification
	Myocardial Infarction	More sensitive tests identifying more people
	High cholesterol 2012	Additional new test

**Table 3:** Mention of possible harms of proposed changes to definitions.

Condition	Panel Comments
ADHD [19]	“main potential negative consequence of raising the age of onset is an increase in prevalence”
Alzheimer disease [30]	“ethical and practical implications” of a “diagnosis” of AD at preclinical stage “need to be studied”
COPD [18]	tests “may lead to more frequent diagnosis of COPD in older adults… as the normal process of aging affects lung volumes and flows, and may lead to under-diagnosis in adults younger than 45”
Diabetes II [22]	need to balance “stigma and costs of mistakenly identifying individuals as diabetic against the minimal clinical consequences of delaying the diagnosis in someone with an A1C level 6.5%”
Mood Disorders panel (Bipolar and Depression) [17]	to prevent “medicalization of normal fluctuations of mood” diagnoses should only be applied when the “clinician determines that the symptoms are associated with clinically significant distress or impairment that require clinical care”
Myocardial infarction [20]	“the current modification of the definition of MI may be associated with consequences for the patients and their families in respect of psychological status, life insurance, professional career…”

Note: for all other panel publications we could identify no mentions.

The average number of panel members was 21 (range, five to 52). Among 15 panels, 12 included members disclosing financial ties to multiple companies, one panel disclosed ties to a single company only (GERD) [21], one stated that members had no relevant conflicts of interest (diabetes II) [22], and one had no disclosure section (high cholesterol 2002) [14], also the oldest panel. For a total of 2,081 individual ties across all categories recorded in the study, there were 55 discrepancies, 2.6%, arising from the independent extraction, mainly involving one or other extractor accidentally missing or adding a specific tie, or making errors by entering a specific tie into an adjacent column or row in a spreadsheet. All were resolved by discussion.

Among 14 panels with disclosure sections, the average proportion of members with industry ties was 75% (range 0%–100%) (Table 4). For members with ties, the median number of pharmaceutical or device companies to which they had declared ties to was seven (Table 4). For the nine panel publications disclosing multiple separate categories of tie, on average, members with industry ties were a consultant/adviser for four companies, received speaker fees/honoraria from two companies, and they or their institutions received research support from three. Twelve panels were chaired or publications led by authors with industry ties, most commonly to multiple companies. Among panels disclosing any ties to government agencies, non-government organisations, or publishers, on average around one-third of panel members disclosed these ties.

**Table 4:** Nature and extent of disclosed ties, by panel.

Panel	Total Number of Industry Ties by Category^a^				Percent Members with Industry Ties	Median *n* (IQR) Companies Tied to	Percent Members with Non-Industry Ties^c^
	Speaker/Honoraria	Consultant/Adviser	Grant/Research	Other^b^			
ADHD 2012 [19]	10	6	9	2	5/9 (56%)	2 (2–5)	7/9 (78%)
Alzheimer disease 2011 [28]–[30]	1	117	23	10	26/46 (57%)	5 (2–7)	6/46 (13%)
Anemia/CKD 2012 [33]	14	34	18	5	15/17 (88%)	3 (2–4)	n/a
Asthma 2009 [15]	67	121	99	6	23/24 (96%)	6 (5–10)	3/24 (13%)
Asthma 2007 [13]	53	103	66	0	16/18 (89%)	7 (5–11)	7/18 (39%)
Bipolar; depression 2012 [17]	17	24	14	6	8/12 (67%)	5 (3–6)	10/12 (83%)
Cholesterol 2012 [16]	25	15	10	0	7/8 (88%)	4 (3–7)	n/a
COPD 2011^d^ [18]	n/a	n/a	n/a	n/a	12/12 (100%)	9 (8–12)	1/12 (8%)
Hypertension 2003 [23]	44	60	62	2	9/11 (82%)	12 (11–13)	3/11 (27%)
MS 2010 [42]	44	104	46	9	17/18 (94%)	7 (5–10)	15/18 (83%)
MI 2012^d^ [20]	n/a	n/a	n/a	n/a	43/52 (83%)	7 (3–12)	11/52 (21%)
RA 2010^d^ [43]	n/a	n/a	n/a	n/a	17/35 (49%)	7 (5–9)	1/35 (3%)

a Individual members can disclose ties to more than one company.

b Other = stock, employee, travel, royalties.

c Non-industry ties include ties to public agencies, non-government organizations, and publishers; some disclosure sections did not include non-industry ties.

d Disclosure sections lumped some categories together.

CKD, chronic kidney disease; IQR, interquartile range; MI, myocardial infarction; MS, multiple sclerosis; n/a, not available; RA, rheumatoid arthritis.

For the 12 panels for which ties were disclosed to more than one company, almost all companies with ties to the three highest proportions of panel members were also active in the market for that panel's condition, with at least one drug on the market or in the research pipeline (Table 5). For example, with the chronic obstructive pulmonary disease [COPD] publication, Astra Zeneca, Boehringer-Ingelheim, and GSK—all companies with drugs for the condition—each had financial ties to 11 of 12 members, including the chair [18]. With the DSM-V Mood Disorders work group, Pfizer and Lilly—with drugs for depression and bipolar—had ties to five of the 12 members [17]. Similarly, companies marketing hypertension drugs—Bristol-Myers Squibb, Merck, Novartis—each had financial ties to eight of the 11 members of the panel which created the new diagnostic category “pre-hypertension” [23].

**Table 5:** Companies with highest proportions of ties, and drugs in therapeutic area.

Panel^a^	Top Companies	*n* and Percent of Panel to Which Company Had Ties	Drug in Therapeutic Area
ADHD [19]	Janssen Cilag	3/9 (33%)	Methylphenidate HCI
	Eli Lilly	2/9 (22%)	Atomoxetine HCI
	McNeil	4/9 (44%)	Methylphenidate HCI
	Shire	2/9 (22%)	Amphetamine (Adderall)
Alzheimer disease [28]–[30]	Pfizer	13/46 (28%)	Donepezil HCI
	Eli Lilly	14/46 (30%)	Solanezumab
	Elan	11/46 (24%)	Bapineuzumab
Anemia/CKD [33]	Amgen	13/17 (76%)	Darbepoetin alfa
	Roche	5/17 (29%)	Methoxy polyethylene glycol-epoetin beta
	Affymax	5/17 (29%)	Peginesatide
	Vifor	4/17 (24%)	Iron supplementation
Asthma 2009 [15]	GSK	20/24 (83%)	Fluticasone propionate
	AZ	19/24 (79%)	Zafirlukast
	Novartis	14/24 (58%)	Omalizumab
Asthma 2007 [13]	AZ	11/18 (61%)	Zafirlukast
	GSK	12/18 (67%)	Fluticasone propionate
	Merck	13/18 (72%)	Montelukast sodium
Bipolar/depression [17]	AZ	3/12 (25%)	Quetiapine fumerate
	Lilly	5/12 (42%)	Duloxetine; olanzapine
	Pfizer	5/12 (42%)	Sertraline HCI; ziprasidone HCI
Cholesterol 2012 [16]	Merck	4/8 (50%)	Simvastatin
	Abbott	3/8 (38%)	Niacin
	AZ	3/8 (38%)	Rosuvastatin
	Novo-Nordisk	3/8 (38%)	n/a
COPD [18]	AZ	11/12 (92%)	Budesonide & formoterol fumarate dihydrate
	BI	11/12 (92%)	Tiotropium bromide
	GSK	11/12 (92%)	Fluticasone propianate
Hypertension [23]	BMS	8/11 (73%)	Irbesartan
	Merck	8/11 (73%)	Losartan
	Novartis	8/11 (73%)	Amlodipine besylate/benazepril hydrochloride
Myocardial infarction [20]	AZ	23/52 (44%)	Rosuvastatin
	Merck	16/52 (31%)	Simvastatin
	Bayer	15/52 (29%)	Rivaroxaban
	BI	15/52 (29%)	Alteplase
Multiple sclerosis [42]	Biogen	13/18 (72%)	Interferon beta-1a
	Merck Serono	12/18 (67%)	Interferon beta
	Sanofi	11/18 (61%)	Teriflunomide
Rheumatoid arthritis [43]	UCB	15/35 (43%)	Certolizumab pegol
	Abbott	14/35 (40%)	Adalimumab
	BMS	13/35 (37%)	Abatacept

a Analysis not possible for Cholesterol 2002, diabetes, GERD panels.

CKD, chronic kidney disease; n/a, not available.

To evaluate any potential impact of the IOM recommendations regarding industry ties, we compared the panel publications released in 2012—after both IOM reports [5],[8]—to those released earlier. We found similar proportions of members disclosing industry ties (76% was the average across 2012 panels; 74% was the average across pre-2012 panels); a small reduction in the median number of companies to which those members disclosed ties in the 2012 panels (four in 2012 panels; seven pre-2012 panels); and similar proportions of panel publications widening definitions (four of six of 2012 publications; six of ten of pre-2012 publications).

## Discussion

In this cross-sectional analysis of panels making recent decisions on definitions of common conditions in the US context, we found most panels proposed widening definitions and most had a majority of members with multiple ties to pharmaceutical companies. Proposals to widen fell into three inter-related categories: creating new categories of “pre-disease”; lowering diagnostic thresholds; and proposing earlier diagnosis or different diagnostic methods (Table 2). In some cases a clear rationale was offered for these changes—as when the hypertension panel cited evidence from original studies and meta-analysis linking normal blood pressure with elevated risks as the reason to create “pre-hypertension” [23]. In other publications, including the 2007 panel proposing changes to the diagnosis of asthma [13], the rational was less clear, more complex and diffuse.

Notwithstanding the problem of under-diagnosis, a growing body of evidence suggests over-diagnosis may be occurring across a range of common conditions, including hypertension [24], asthma [25], attention deficit hyperactivity disorder (ADHD) [26], and COPD [27]. Yet less than half of the study publications mentioned potential harms of proposed changes to definitions, and none included a rigorous evidence-informed discussion of those risks or how they might be mitigated.

In a three-part publication in 2011 [28]–[30] proposing new categories of “pre-clinical” Alzheimer disease (for research only at this stage) and “predementia”—which would clearly expand the population labelled—there was one short reference to the need to study the “ethical and practical implications” of diagnosing people at a “preclinical” stage [30]. The panel proposing changes to assessment and classification of COPD briefly mentioned that diagnostic methods “may lead to more frequent diagnosis of COPD in older adults with mild COPD as the normal process of aging affects lung volumes and flows” [18], but did not explicitly refer to the risk of “over-diagnosis” as it had done in a previous version of its report [31]. Proposing changes to ADHD diagnostic criteria—in part to make the condition more amenable to being a “lifespan” disorder involving adults as well as children—the DSM-V panel mentioned potential increases in prevalence but suggested they would be “negligible” (Table 3) [32].

Among panels disclosing ties, almost all chairs had financial ties to industry, and an average of three-quarters of members had ties to a median of seven companies, commonly working as consultants, advisers and/or speakers, as well as receiving research support. Companies with financial relationships with the greatest proportion of panel members were marketing or developing drugs for the same conditions about which those members were making critical judgements. GSK for example, marketing top-selling products for asthma, had financial ties to 20 of the 24 members of the 2009 asthma panel, and all 20 were consultant/advisers and/or declared speaker/honoraria ties to GSK [15].

This study has several important limitations. First, the lack of a comparison group means it is impossible to draw any inference of association between frequency of industry ties and proposals to change disease definitions. The exclusion of common conditions too broad to enable a focussed analysis of single panel publications (e.g., back problems) means it may have missed potentially important examples of changing disease definitions and limits generalizability of findings. The focus on the United States—chosen explicitly because of its globally influential panels such as DSM-V workgroups—also limits generalizability. A fourth limitation is reliance solely on disclosed ties, likely leading to an underestimate of their extent. Finally, we note that while we tried to ensure an exhaustive and multi-layered search strategy, we are unaware of any established method for identifying panel publications that review or propose changes to disease definitions.

Notwithstanding these limitations, the study has strong clinical, research, and policy relevance. Its novel focus on panels reviewing and proposing changes to common disease definitions or diagnostic criteria will help deepen understanding of the nature of what's been described as the “modern epidemic” of over-diagnosis [2]. Moreover, the group of 16 publications includes influential articles affecting the definition of 14 common conditions and impacting directly on medical policy and practice around the world.

The study findings are consistent with and help augment the evidence-base about industry ties of influential medical professionals. The 2011 systematic review found 56%–87% of clinical guideline writers had conflicts of interest [6], similar to our finding of 75% across disease-defining panels. Kung and colleagues found 71% of guideline committee chairs had conflicts [9], again similar to our findings. While these proportions may reflect the level of ties among medical specialists more generally, they are in stark contrast to IOM 2009 and 2011 reports calling for panels to generally exclude people with conflicts of interest [5],[8]. As reported above, we found no change in the proportion of members disclosing ties in the 2012 publications, after release of both IOM reports.

At least two publications [20],[21] made reference to members believing industry ties did not influence their decision-making, and we make no suggestion to the contrary. Indeed our data do not support any inference industry ties are associated with widening definitions or failure to rigorously assess potential harms of that widening. With anemia in chronic kidney disease, a panel with a high proportion of ties raised thresholds, effectively narrowing the definition [33]. There will doubtless be other cases where diseases have been widened by panels of medical specialists without industry ties. Moreover, as Lurie and colleagues found in the context of drug regulation, the financial conflicts of expert advisory committees did not correlate significantly with their voting outcomes [34]. Medicalization and over-diagnosis are driven by many factors—technological, professional, commercial, legal, and cultural [3].

While inferences of association or causation between industry ties and expanding disease definitions cannot be drawn, our findings can be considered in the context of broader evidence about potentially distorting biases associated with widespread industry sponsorship and financial ties in medical research [35]–[37], education [38], and practice [5], and in relation to “key opinion leaders” who speak and consult for industry [39].

In 1999 Schwartz and Woloshin found changes to definitions of high blood pressure, high cholesterol, diabetes, and overweight would “dramatically inflate disease prevalence” and “ultimately label 75% of the adult U.S. population as diseased” [40]. They concluded the “extent to which new ‘patients’ would ultimately benefit from early detection and treatment of these conditions is unknown. Whether they would experience important physical or psychological harm is an open question.” To what extent newly created “patients” produced by widening disease definitions will experience important harms remains a largely unanswered question, over a decade later.

This study did not investigate the merits of the proposed changes to the conditions identified. However, findings that diagnostic thresholds are being lowered by panels dominated by those with financial ties to multiple companies that may benefit directly from those decisions raise questions about current processes of disease definition. While it may be more difficult to locate senior specialists without industry ties, two recent IOM reports have encouraged such a change [5],[8], and models already exist for panels free of such conflicts, including the NIH consensus development program [41].

Several unanswered questions arise from this study, which could benefit from further investigation. Researchers might fruitfully examine how definitions are changing over time, what dollar amounts are being received from industry by panel members and organisations that auspice them, and how panel proposals impact on potential markets of sponsors. Most importantly enhanced research and policy attention might be directed at designing new processes for reviewing disease definitions, free of financial conflicts of interest and informed by rigorous analysis of benefits and harms.

## Supporting Information

Table S1 **Asthma search strategy.**
(DOCX)

## Abbreviations

ADHD: attention deficit hyperactivity disorder
COPD: chronic obstructive pulmonary disorder
DSM: Diagnostic and Statistical Manual of Mental Disorders
GERD: gastroesophageal reflux disease
IOM: Institute of Medicine
NIH: National Institutes of Health
