# Supplementary material for: Expanding Disease Definitions in Guidelines and Expert Panel Ties to Industry: A Cross-sectional Study of Common Conditions in the United States
Source: PLoS Med. 2013 Aug 13;10(8):e1001500. doi: 10.1371/journal.pmed.1001500 (PMC3742441; doi:10.1371/journal.pmed.1001500)
Supplement: Table S1 — Asthma search strategy. (DOCX) [file pmed.1001500.s001.docx]

**Supplemental Table S1: Asthma Search Strategy**

Database: Ovid MEDLINE(R) <1946 to July Week 2 2012>

Search Strategy: Asthma

--------------------------------------------------------------------------------

1 consensus/ (3929)

2 exp guideline/ (22675)

3 (consensus or report or recommend* or "position paper" or statement or guidance or guideline*).ti. (397131)

4 or/1-3 (407005)

5 ((expert* or advisory or scientific or review or national or working or professional or governing) adj3 (panel or group or meeting or conference or committee or board or agenc* or body)).tw. (50995)

6 (taskforce or "working party" or association or societ*).tw. (774846)

7 5 or 6 (816637)

8 4 and 7 (36140)

9 exp *Asthma/ (80805)

10 exp Asthma/cl, di [Classification, Diagnosis] (13608)

11 (asthma* and (class* or diagnos* or defin*)).tw. (21202)

12 asthma.ti. (56667)

13 or/9-12 (92056)

14 8 and 13 (314)

15 limit 14 to (english language and yr="2000 -Current") (154)
